# HDL-IDS: A Hybrid Deep Learning Architecture for Intrusion Detection in the Internet of Vehicles

**DOI:** 10.3390/s22041340

**Published:** 2022-02-10

**Authors:** Safi Ullah, Muazzam A. Khan, Jawad Ahmad, Sajjad Shaukat Jamal, Zil e Huma, Muhammad Tahir Hassan, Nikolaos Pitropakis, Arshad, William J. Buchanan

**Affiliations:** 1 Department of Computer Science, Quaid-i-Azam University, Islamabad 44000, Pakistan; safiullah@cs.qau.edu.pk (S.U.); muazzam.khattak@qau.edu.pk (M.A.K.); 2 Pakistan Academy of Sciences, Islamabad 44000, Pakistan; 3 School of Computing, Edinburgh Napier University, Edinburgh EH10 5DT, UK; n.pitropakis@napier.ac.uk (N.P.); b.buchanan@napier.ac.uk (W.J.B.); 4 Department of Mathematics, College of Science, King Khalid University, Abha 61413, Saudi Arabia; shussain@kku.edu.sa; 5 Department of Electrical Engineering, Institute of Space Technology, Islamabad 44000, Pakistan; zilehuma@ist.edu.pk; 6 Department of Mechanical Engineering, Bahauddin Zakariya University, Multan 66000, Pakistan; tahirqureshi@bzu.edu.pk; 7 Institute for Energy and Environment, University of Strathclyde, Glasgow G1 1XQ, UK; arshad.100@strath.ac.uk

**Keywords:** deep learning, gated recurrent units, Internet of Things, Internet of Vehicles, long short-term memory, machine learning

## Abstract

Internet of Vehicles (IoV) is an application of the Internet of Things (IoT) network that connects smart vehicles to the internet, and vehicles with each other. With the emergence of IoV technology, customers have placed great attention on smart vehicles. However, the rapid growth of IoV has also caused many security and privacy challenges that can lead to fatal accidents. To reduce smart vehicle accidents and detect malicious attacks in vehicular networks, several researchers have presented machine learning (ML)-based models for intrusion detection in IoT networks. However, a proficient and real-time faster algorithm is needed to detect malicious attacks in IoV. This article proposes a hybrid deep learning (DL) model for cyber attack detection in IoV. The proposed model is based on long short-term memory (LSTM) and gated recurrent unit (GRU). The performance of the proposed model is analyzed by using two datasets—a combined DDoS dataset that contains CIC DoS, CI-CIDS 2017, and CSE-CIC-IDS 2018, and a car-hacking dataset. The experimental results demonstrate that the proposed algorithm achieves higher attack detection accuracy of 99.5% and 99.9% for DDoS and car hacks, respectively. The other performance scores, precision, recall, and F1-score, also verify the superior performance of the proposed framework.

## 1. Introduction

Internet of Things (IoT) is an advanced technology that connects smart devices to the internet, such as the Internet of Vehicles (IoV), wireless cameras, and other electronic devices. Due to the rapid increase of connected vehicles, several security and privacy challenges have been introduced [1,2,3]. A basic framework for communications between vehicular networks is IoV [4]. It establishes a dependable network transmission between vehicles [5]. The IoV network consists of two sub-networks—intra-vehicle network and inter-vehicular network. The intra-vehicle network involves internal electronic devices and sensors of a vehicle, which are connected to a centralized controller for message transmission and performing a specific task [6]. While an inter-vehicular network connects a vehicle to external devices using vehicle-to-everything (V2X) technology. V2X allows communication between vehicles and other communicative devices, such as signal antennas and other roadside infrastructure [7,8].

The security risks increase with the rapid growth in the connectivity of smart vehicles. An attack on the IoV network can affect stability, reliability, and cause accidents in vehicles. In June 2021, the World Health Organization (WHO) stated that every year 1.3 million deaths occur due to car accidents [9]. In a real-life example, two hackers hacked a vehicle, took control of steering and brakes, and performed dangerous actions at high speed [10].

During a cyber attack on a vehicle network, the attacker takes control of a vehicle, where he/she can perform dangerous stunts. A hacker has the ability to disable the brakes or jerk the steering wheel at a high speed, which may potentially lead to an accident. The attacker can also carry out a distributed denial of service (DDoS) attack, which engages the car controller area network (CAN) bus and prevents IoV-based vehicles from accessing the brakes at crucial times [11,12]. DDoS attacks on inter-vehicle networks keep channels busy, such as not letting traffic signal lights turn red and keeping them green in dangerous places that may lead to accidents [13].

An intrusion detection system (IDS) is needed to monitor network traffic and detect malicious attacks. The performance of IDS depends on the accuracy of the detection algorithm. Improving the accuracy of IDS will reduce the false alarm rate. Existing IDSs have difficulty in improving performance and detecting unknown attacks. Machine learning (ML) techniques provide automated detection systems with impressive performance. Moreover, ML techniques have general capabilities to detect unknown attacks. Deep learning (DL) is a branch of ML, whose performance is remarkable. On the basis of performance, DL methods have become a research “hotspot” [14,15,16].

**Figure 1:**
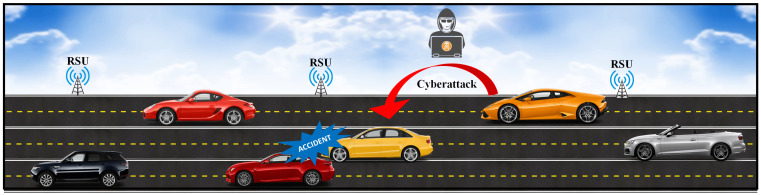
A cyber attack scenario in smart vehicle.

The purpose of IDS is to identify different types of malicious network traffic and computer activities that a regular firewall might miss [17]. From a trained set, ML can learn essential details. Moreover, ML algorithms handle nonlinear data and are easy to train [18,19,20]. A generic cyber attack scenario on smart vehicles is presented in Figure 1. Several researchers have suggested ML techniques for reducing issues related to smart vehicles. A proficient and fast algorithm is needed to detect malicious attacks in IoV. DL algorithms provide more efficient performances than traditional ML algorithms [21,22,23]. For IDS, some commonly used DL algorithms are convolutional neural network (CNN), recurrent neural network (RNN), LSTM, and GRU. The CCN is more complex than other DL algorithms, because it requires data-like images in matrix form; the data must be normalized and converted into the form of an image matrix [24,25,26,27,28,29]. The LSTM and GRU algorithms are effective at detecting malicious assaults over other ML and DL algorithms. Moreover, in IoV, some vehicles are connected for long time periods in which conventional ML models fail to convey long-term results. LSTM and GRU algorithms provide good accuracy in detecting malicious attacks [30].

For better performance, every DL algorithm requires more than one layer. The LSTM performance on multiple layers is much better than GRU, but the training and response time is high, while GRU training and response time are better than LSTM, but performance is low [31]. Improving GRU performance and reducing the response time of LSTM in multi-layers, this paper presents an HDL-IDS scheme that combines LSTM and GRU algorithms. This hybrid combination can provide better performance in terms of accuracy and response time.

Multiple real-time datasets available are generated by different researchers for the detection of malicious attacks. Some old datasets have data for old attack detections, and some new datasets have data for new attack detections. In this paper, we used two datasets, a combined DDoS of CIC DoS, CI-CIDS 2017, and CSE-CIC-IDS 2018 for detection of DDoS attacks in inter-vehicular networks, and a car-hacking dataset for the detection of DDoS, fuzzing, and spoofing in an intra-vehicular network [32,33].

### Contribution

- This paper suggests pre-processing techniques that include cleaning, shuffling, feature filtering, and normalization. In pre-processing, the shuffling technique applies to the dataset that shuffles the dataset in random fashion for training and testing of the model and improves the performance of the model.
- A novel hybrid technique of LSTM-GRU is presented for intrusion detection in IoV.
- The proposed approach reduces the training and response time and significantly improves the attack detection accuracy.

The remaining paper is organized as follows. Section 2 presents some of the latest research related to the intrusion detection in IoV. Section 3 comprises mathematical modeling and overall flow of the proposed architecture. Section 4 discusses the experimental methodology. Section 5 presents a brief discussion on experimental findings of the proposed model. Finally, a brief conclusion is presented in Section 6.

## 2. Related Work

This section presents some of the latest research contributions related to intrusion detection in IoV. Ashraf et al. [34] presented a DL-based IDS for intelligent transportation systems (ITS) that learns the behaviour of regular network traffic in an intra-vehicle network, V2V communications, and V2I networks. The proposed IDS is based on the LSTM autoencoder, which recognize anomalous events in IoV from the main gateway. The evaluation of the model was done with the car-hacking and UNSWNB15 datasets for intra-vehicle and inter-vehicle communications. Injadat et al. [35] proposed a novel multi-stage optimized ML-based model for detection of cyber attacks. The main purpose of this model was to reduce the computational power and provide better performance of the system. Researchers evaluated the performance of the proposed scheme using CICIDS2017 and UNSW-NB15 datasets.

In another study, Zaidi et al. [36] applied statistical methods to examine the flow of IoV traffic to find rogue and more malicious nodes. In this technique, the flow of network traffic was first collected and then intrusion detection was assessed. The proposed IDS can decide to approve or disapprove the coming data on the basis of traffic flow insight analysis. The performance of this method is better for detecting rogue and more malicious nodes. Whenever multiple malicious events occur, the accuracy of this method becomes low. Anbalagan et al. [37] proposed a memetic-based RSU (M-RSU) model for fast communications in a wide area. Researchers also proposed a distributed ML (DML) model for the detection of attacks in the IoV network. Nie et al. [38] proposed a traditional CNN model to extract the features from RSU and detect the attacks in IoV. Olufowobi et al. [39] developed an effective algorithm to estimate the real-time arguments of response time analysis (RTA) model using a black box technique. They presented the SAIDuCANT IDS paradigm, which specifies desired the behavior of the CAN bus, and then identifies violations as indicators of a negotiated network. Researchers discussed two new measures, time to detection (TD) and false positives before attack (FPBA), which assess an IDS performance, for which SAIDuCANT outperforms existing detection algorithms in terms of accuracy and consistency.

Zhang et al. [40] generated their own dataset from a real-time vehicular network and proposed an ANN model for IDS in intra-vehicle networks. The proposed algorithm improved the accuracy of IDS up to 98% by using gradient descent with momentum (GDM), and GDM with adaptive gain. Kang et al. [41] developed a deep learning paradigm for IDS in intra-vehicle networks. The main purpose of the paradigm was to improve the accuracy of IDS. Researchers worked on binary classifications that were benign and assault data. For the classification, they calculated the probability of each packet to classify it is an anomaly or normal packet. Researchers utilized their own generated datasets for training and testing of the model. Comparisons of the existing study with state-of-the-art models are shown in Table 1 and Table 2. These tables show the existing models with various features in the related work. The proposed study includes missing features of previous models.

## 3. The Proposed Hybrid Deep Learning Model for Intrusion Detection in IoV

Intrusion in IoV is very dangerous to human life. An attack on inter-vehicular networks can disturb the communication between smart vehicles. The vehicle cannot get any information about road situations. Moreover, an intra-vehicular network is more sensitive than an inter-vehicular network because, in a vehicle, the main target of the attacker is the CAN bus. A black hacker can attack the CAN bus of a vehicle that takes control of the internal main controller of the vehicle, which may cause an accident. To protect human life, it is essential to deploy the security firewalls against these types of cyber attacks. Several researchers suggested ML techniques to reduce issues related to smart vehicles. For multiple layer models, many researchers used LSTM or GRU or combined it with other DL algorithms for the improvement of performance on the detection rate of malicious attacks in IoV, but the response time of the system becomes high. LSTM and GRU work on time series data and always learn from previous time steps.

**Figure 2:**
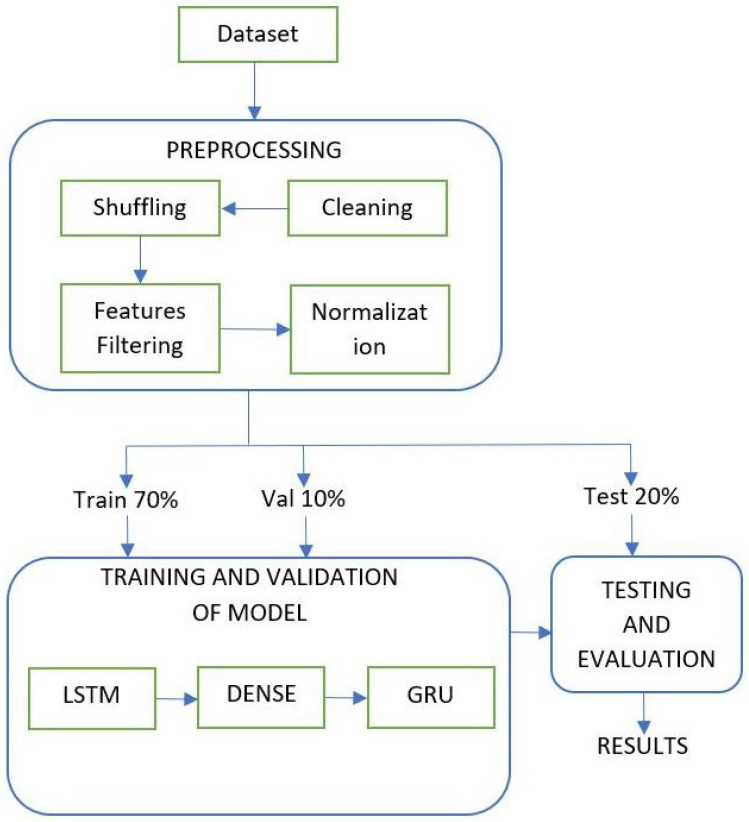
Block diagram of the proposed model for intrusion detection in IoV.

The vanishing gradient problem of RNN is addressed by the LSTM and GRU. The performance of multiple layer LSTM for intrusion detection is better, However, the response time is high. Moreover, the response time of GRU is less, but the performance is not as good as LSTM [31]. In this paper, we propose a hybrid DL model that combines LSTM and GRU. The block diagram of the proposed architecture is presented in Figure 2. This framework contains three layers—LSTM, DENSE, and GRU. The proposed model reduces the training and response times of multiple layers on LSTM and gives better performance in detecting malicious attacks in IoV.

### 3.1. LSTM

**Figure 3:**
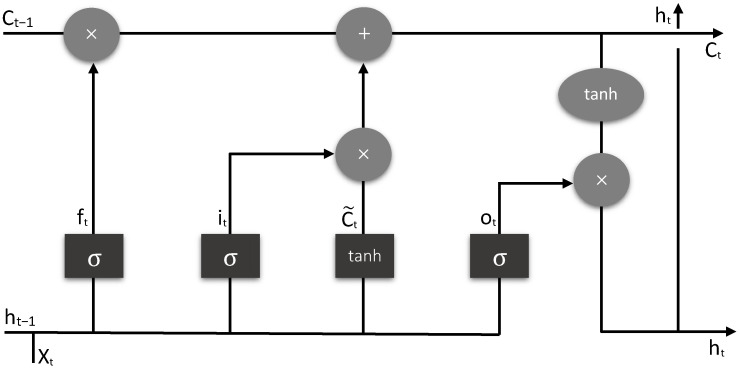
Basic architecture of LSTM.

LSTM is the first hidden layer of the proposed model. The input to the first hidden layer is given as (none, 48, 1) for the combined DDoS dataset, and (none, 10, 1) for the car-hacking dataset. Here, “none” is the dynamic size number of instances, “48” is the number of features, and “1” is the third dimension value. The output shape of this layer is (none, 48, 24) for combined DDoS and (none, 10, 20) for the car-hacking dataset, which is the input to the next layer. LSTM has a series of gates for the flow control of information, for example, how data come in, saves it, and leaves. Moreover, there are two more states—cell state and hidden state. Typically, LSTM has five activation functions, three sigmoid functions (one in each gate), and two Tanh functions (one with the input gate and the second with output gate). Mainly, there are three gates in LSTM—forget, input, and output gates, as shown in Figure 3. The forget, input, and output gates are mathematically described in Equations (1)–(3), respectively.

(1) $$f_{t}=\sigma \left(w_{hf}h_{t-1}+w_{xf}x_{t}+b_{f}\right)$$

(2) $$i_{t}=\sigma \left(w_{hi}h_{t-1}+w_{xi}x_{t}+b_{i}\right)$$

(3) $$o_{t}=\sigma \left(w_{ht}h_{t-1}+w_{xt}x_{t}+b_{t}\right)$$

The new hidden state value is calculated by using Equation (4) and the cell state value by using Equations (5) and (6). The input, output, and forget gates are represented by ‘*i*’, ‘*o*’, and ‘*f*’, respectively. ‘*w*’ represents weight, ‘*h*’ represents hidden state, ‘*x*’ represents the input data, ‘*b*’ represents bias, and ‘*C*’ represents cell state.

(4) $${\tilde{C}}_{t}=\tanh\left(x_{t}w_{xg}+w_{g}h_{t-1}+b_{g}\right)$$

(5) $$C_{t}=\sigma \left(f_{t}*C_{t-1}+i_{t}x_{t}*{\tilde{C}}_{t}+b_{c}\right)$$

(6) $$h_{t}=\tanh\left(C_{t}+b_{h}\right)*o_{t}$$

### 3.2. DENSE

DENSE is the second layer in the proposed model to join LSTM with GRU, and gives quick responses. The DENSE layer gets the values from the previously hidden layer in the (none, 48, 24) shape for the combined DDoS dataset and the (none, 10, 20) shape for the car-hacking dataset. This layer is connected to the previous layer. The output shape of this layer is (none, 48, 12) and (none, 10, 10) for the above-mentioned datasets, respectively, which is the input to the next layer. We used the rectified linear activation unit (ReLU) in this layer. The ReLU activation function worked on positive values. In this experiment, the positive values are between 0 and 1. The speed of the ReLU function is faster than other activation functions and it also reduces the vanishing gradient problem. The ReLU is described in Equation (7).

(7) $$R_{x}=\max\left(0,x\right)$$

### 3.3. GRU

**Figure 4:**
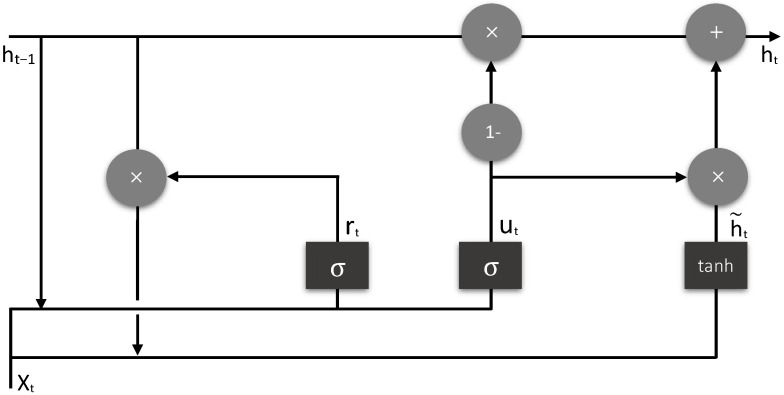
Basic architecture of GRU.

GRU is the third layer in the proposed model that takes values from the previous DENSE layer and produces the final output. The GRU layer gets the values from the previous layer in the (none, 48, 12) shape for the combined DDoS dataset and the (none, 10, 10) shape for the car-hacking dataset. This layer produces the output probability. GRU has two gates—reset gate and update gate, and one hidden state. There are two sigmoid activation functions in GRU (one in each gate) and one Tanh function for the output shown in Figure 4. For multi-class detection, the softmax activation function is used. The reset and updated gates are described in Equations (8) and (9), respectively.

(8) $$r_{t}=\sigma \left(\left(w_{xr}x_{t}+w_{hr}h_{t-1}+b_{r}\right)\right)$$

(9) $$u_{t}=\sigma \left(\left(w_{xu}x_{t}+w_{ur}h_{t-1}+b_{u}\right)\right)$$

The new hidden state value has calculated using Equations (10) and (11). The reset and update gates are represented by ‘*r*’ and ‘*u*’, respectively.

(10) $$\left.{\tilde{h}}_{t}=\tanh\left(w_{hx}x_{t}+w_{h}h\left(r_{t}h_{t-1}\right)+b_{u}\right)\right.$$

(11) $$h_{t}=\left(1-u_{t}\right)h_{t-1}+u_{t}{\tilde{h}}_{t}$$

## 4. Experimental Methodology

In this section, a detailed experimental methodology is presented. This section contains dataset description, preprocessing, and feature selection process.

### 4.1. Datasets

There are multiple real-time network datasets available for intrusion detection, such as DARPA, NSL-KDD, CIC DoS dataset 2016, CICIDS 2017, CSE-CIC-IDS 2018 AWS, and CIC-DDoS 2019. For this experiment, we used two datasets—a combined DDoS dataset for the inter-vehicular network and a car-hacking dataset for the intra-vehicle network, as shown in Table 3.

#### 4.1.1. Combined DDoS Dataset

The combined DDoS dataset was created from the combination of real-time network DDoS datasets—CIC DoS 2016, CICIDS 2017, and CSE-CIC-IDS 2018. This dataset consists of two classes of data, DDoS and Benign. The number of DDoS records in the dataset was 6,472,647 and the number of Benign records was 6,321,980. CIC DoS 2016, CICIDS 2017, and CSE-CIC-IDS 2018 datasets included inter-vehicle network flow data according to DDoS attacks [5,32]. CIC DoS 2016 dataset included slowbody2, ddosim, goldeneye, hulk, slowloris, rudy, and slowread attacks. CICIDS 2017 dataset included DDoS-LOIC and port scan attacks. The CSE-CIC-IDS 2018 dataset included SlowHTTPTest, hulk, slowloris, LOIC-types of DDoS attacks. A collection of these datasets, with identical features, included the different types of DDoS attacks in a single combined dataset [32]. The combined dataset includes different types of DDoS attacks that are found in inter-vehicular networks. The inter-vehicular network can suffer from port scan, DDOSIM, goldeneye, hulk, slowloris, rudy, slowread, SlowHTTPTest, or LOIC-types of DDoS attack, which are included in the combined dataset [42,43].

#### 4.1.2. Car-Hacking Dataset

The car-hacking dataset was generated for the detection of cyber attacks in the internal network of the vehicle. This dataset mainly works on the CAN bus, which can target the attacker [33]. There are four different files—DDoS, Fuzzy, gear, and RPM, in which gear and RPM are spoof attack files. In each file of the dataset, the classes are represented with R and T, which represent benign and malicious values, respectively. In this experiment, first, we renamed each file label with the name of the class, such as DDoS, Fuzzy, Spoof, and Benign, and then combined all of these files into a single data frame.

### 4.2. Cleaning

Each dataset has multiple records. A dataset must be checked before training the model for empty and undefined records. For the cleaning, we used python libraries (Pandas and NumPy) and functions to check the dataset for missing and infinite values, which returned Boolean values, true or false. True meant there were some missing or infinite values and false meant the dataset was clean. In our experiments, two datasets were utilized that had some undefined and empty records. To clean these datasets, all undefined records were converted into empty records. After conversion of undefined values to empty, all empty records were removed from the datasets.

### 4.3. Shuffling

This technique is used to shuffle dataset tuples. In our experiments, the combined balance DDoS dataset was used for the inter-vehicular network that had two classes—benign and DDoS. The second was the car-hacking dataset, which was used for intra-vehicle networks. Data in the combined dataset were arranged in a sequential form, such as complete data of one class then complete data of the second class. The training and testing of the model required both types of data. To improve the performance of the model, and for better testing, it required shuffled data. The shuffling method was used to shuffle all of the data in a random fashion.

### 4.4. Feature Filtering

Every dataset has a number of features. If a dataset has multiple features and also has some unimportant features that cannot affect the output label, then we must remove it from the dataset, because, it produces overfitting and underfitting, which will affect the time complexity and performance of the model. Feature selection is a technique used to remove unimportant features from the dataset and leave only important features. The main goal of feature selection was to prevent overfitting, underfitting, to improve the performance, and reduce the training time and response time of the model.

**Figure 5:**
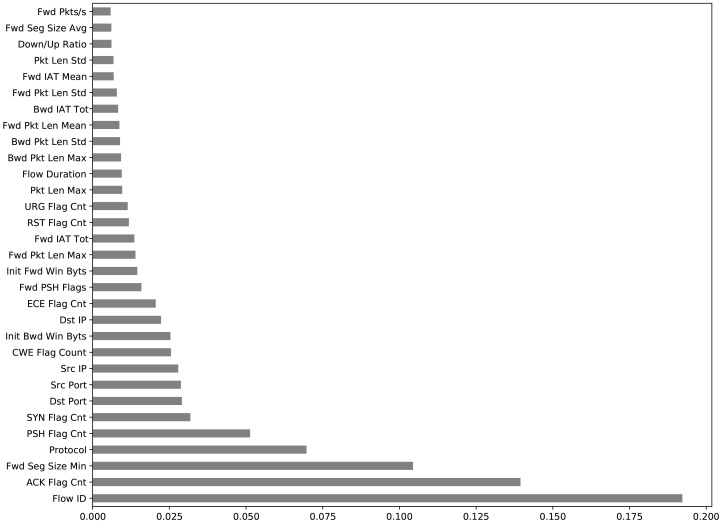
Combined dataset ranked features.

**Figure 6:**
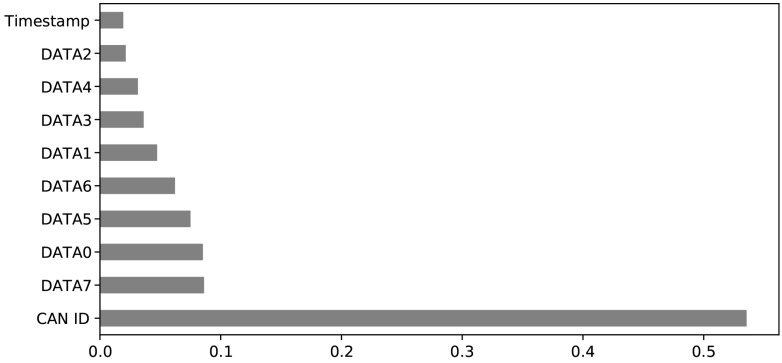
Car-hacking dataset ranked features.

When we trained the proposed model with all 84 features of the combined dataset and all 12 features of the car-hacking dataset, the performance of the model declined and gave 49.71% and 82.35% accuracies, respectively. To improve the performance of the model, the features of the dataset have to be reduced and the unimportant features have to be eliminated. There are mainly three methods for feature selection—wrapper, filter, and intrinsic methods. In this work, the filter method was used. The extra tree classifier (ETC) method was used in the feature filtering. This method ranks all features according to their entropy, as shown in Figure 5 and Figure 6. All features with values greater than 0.0017 were selected. After ranking removed the unimportant and very low-rank features that could not affect the output class, the remaining important features were 48; one was the label of the combined dataset and 10 important features, and one was the label of the car-hacking dataset.

### 4.5. Balancing Strategies

Each dataset contains multiple records. Before training and testing the model, make sure that each class has the same number of instances or slight variations. There are basically two ways to equalize the number of instances in each class—undersampling and oversampling. Undersampling can potentially remove important instances, and random oversampling can replicate the same instances that can cause overfitting.

In this experiment, the combined DDoS dataset was balanced, but the car-hacking dataset was unbalanced. There were four classes of data in four different files. Each file contained normal and injected data. We selected the injected instances from each file, and for normal instances, used a normal file. Separate numeric labels for each class was assigned and then all were into one data frame. To balance the dataset, the synthetic minority oversampling technique (SMOTE) was used to create artificial instances. SMOTE uses the K-nearest neighbor (KNN) method to select a very near-random instance. SMOTE generates new instances inside the same class range. For creating training, validation, and testing sets, a stratified random sampling (SRS) technique was used. The SRS technique takes an equal number of instances from each class and creates train, test, and validation sets.

### 4.6. Normalization

Normalization is a technique to scale the numeric values between the common scale ranges and remove problems from the dataset, such as different values in different features; for example, one feature values between 0 and 1 and the other feature values 100 and 1000, so it can affect the training of the model. In our experiments, the dataset has very large values in some features and very small values, such as negative values. To address this problem, firstly, the categorical features were converted into numerical values. Each feature had multiple categories, for which one-hot-encoding required larger memory and was time-consuming [44]. In this experiment, we used the label encoder technique for the conversion of categorical attributes to numerical. After the conversion, we used the min–max normalization technique to normalized values between 0 and 1 by using Equation (12).

(12) $$X_{\mathrm{norm}\;}=\frac{x-x_{\min}}{x_{\max}-x_{\min}}$$

## 5. Simulations and Results

In this section we discuss the results obtained from the experiments. We conducted two experiments on the proposed model. The first experiment was conducted for the inter-vehicular networks, which was for binary class detection DDoS and benign. The DDoS attack is famous for bringing the whole network down. This is more dangerous than other attacks because DDoS spreads rapidly in the network and causes sufficiently higher damage. DDoS attack on the network overwhelm the channel. In this process, network traffic is flooded on a targeted server, which results in the network not working properly or malfunctioning. In such circumstances, it becomes difficult for a vehicle to send/get critical information [45,46].

The second experiment was performed for the intra-vehicular network multi-class attack detection on a CAN bus. The evaluation of the model for the inter-vehicular network was performed by using a combined dataset. For intra-vehicle attack detection, multi-class classification was performed by using the car hack2020 dataset. The proposed scheme was tested on Adam, Nadam, and Adamax optimizers with batch size 32, and probabilistic loss functions. The binary cross-entropy function was used for the combined DDoS dataset and a sparse categorical cross-entropy function for car hack2020 dataset.

### 5.1. Experimental Setup

The proposed scheme was implemented by using an Intel Core i5 8th generation laptop. All experiments were performed in a Python 3.0 simulation environment to analyze the performance of the proposed model.

### 5.2. Evaluation Measure

Evaluation is a technique that measures the performance of the model. For the evaluation, several researchers use precision, recall, F1-measure, and accuracy. The evaluation of the proposed model was done with 20% data of a combined DDoS dataset for an inter-vehicular network and 20% data of a car hack2020 dataset for the intra-vehicular network. To evaluate the performance of the proposed scheme, a number of evaluation metrics were utilized, including accuracy, precision, recall, and F1-score. The evaluation metrics are calculated with true positive (*TP*), true negative (*TN*), false positive (*FP*), and false negative (*FN*). All of these performance scores can be measure by using Equations (13)–(16).

(13) $$\;\mathrm{Accuracy}\;=\frac{TP+TN}{TP+TN+FP+FN}$$

(14) $$\;\mathrm{Precision}\;=\frac{TP}{TP+FP}$$

(15) $$\;\mathrm{Recall}\;=\frac{TP}{TP+FN}$$

(16) $$F1-\mathrm{measure}=\frac{2*(\;\mathrm{Pression}\;*\;\mathrm{Recall}\;)}{\;\mathrm{Pression}\;+\;\mathrm{Recall}\;}$$

### 5.3. Inter-Vehicular Network

**Figure 7:**
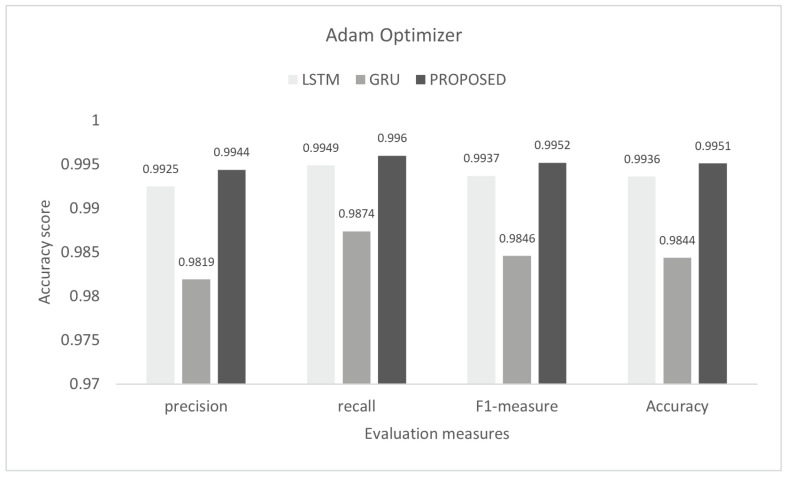
Evaluation on Adam with the combined DDoS dataset.

**Figure 8:**
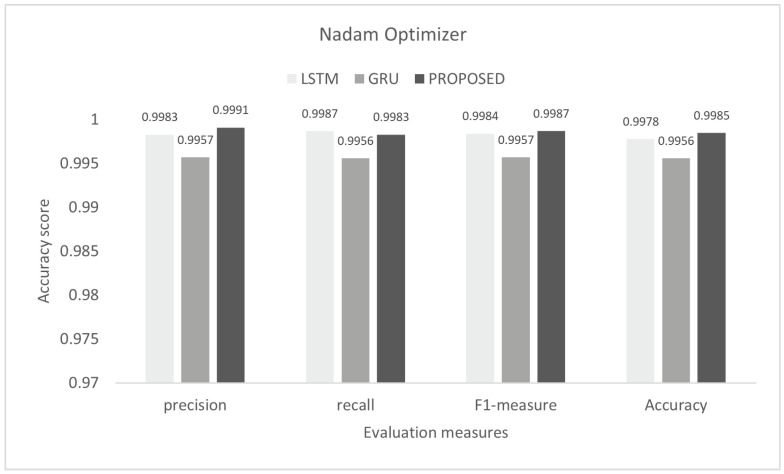
Evaluation on Nadam with the combined DDoS dataset.

**Figure 9:**
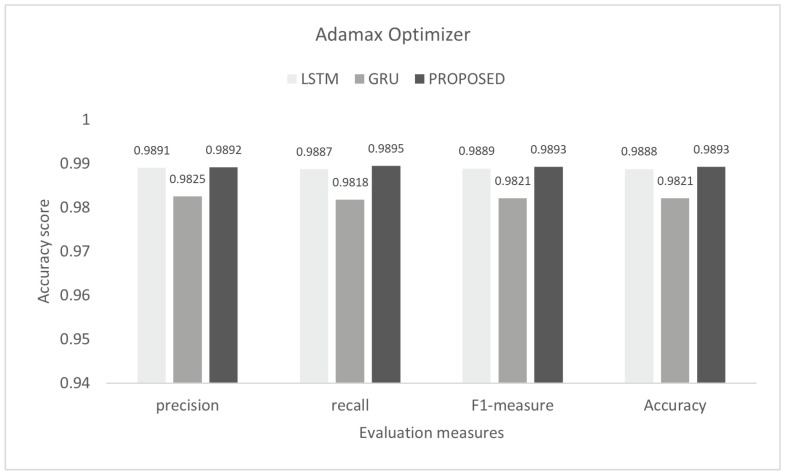
Evaluation on Adamax with the combined DDoS dataset.

**Figure 10:**
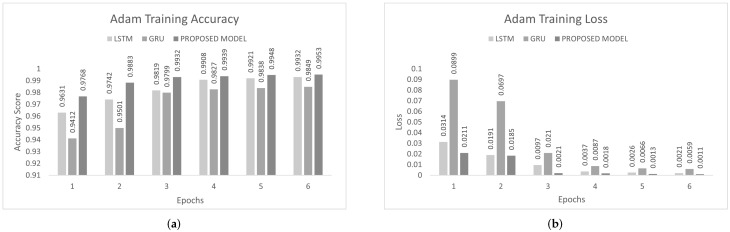
Adam training on the combined DDoS dataset. (**a**) Adam training accuracy on the combined dataset, (**b**) Adam training loss on the combined dataset.

**Figure 11:**
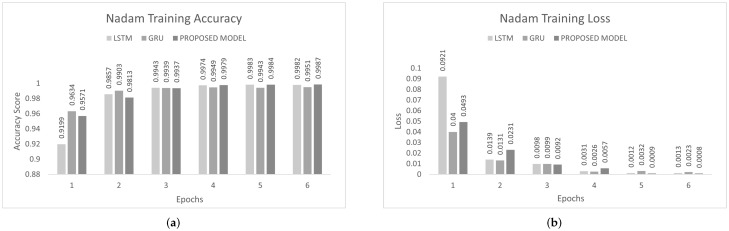
Nadam Training on Combined DDoS dataset. (**a**) Nadam Training Accuracy on Combined dataset, (**b**) Nadam Training Loss on Combined dataset.

**Figure 12:**
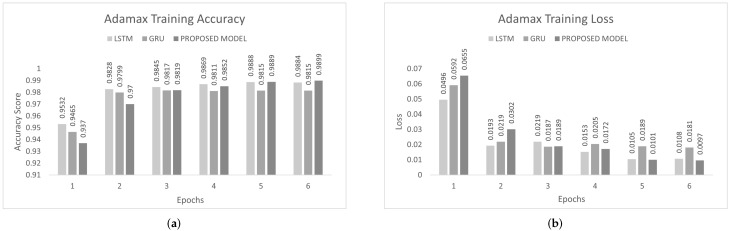
Adamax training on the combined DDoS dataset. (**a**) Adamax training accuracy on the combined dataset, (**b**) Adamax training loss on the combined dataset.

The inter-vehicular network enables vehicles to communicate with each other and roadside infrastructure. The attacker can target the network and stop the communication of the inter-vehicular network. In an inter-vehicle network, the attacker can easily launch a DDoS attack due to mobility. All services of the network will be stopped by the DDoS attack [47]. We conducted the experiment on the proposed model to detect cyber attacks in the inter-vehicular network by using a combined DDoS dataset. The proposed model was the combination of LSTM and GRU with the DENSE ReLU layer. The training process was conducted for six epochs. In this experiment, we used the binary cross-entropy function for loss. The proposed scheme was tested on Adam, Nadam, and Adamax optimizers. Adam gave 99.44% precision, 99.60% recall, 99.52% F1-measure, and 99.51% accuracy. Nadam gave 99.91% precision, 99.83% recall, 99.87% F1-measure, and 99.85% accuracy. Adamax gave 98.92% precision, 98.95% recall, 98.93% F1-measure, and 98.93%. The comparison with LSTM and GRU is shown in Figure 7, Figure 8 and Figure 9. The training accuracy and loss on the Adam optimizer is shown in Figure 10, Nadam optimizer is shown in Figure 11, and Admax is shown in Figure 12.

**Figure 13:**
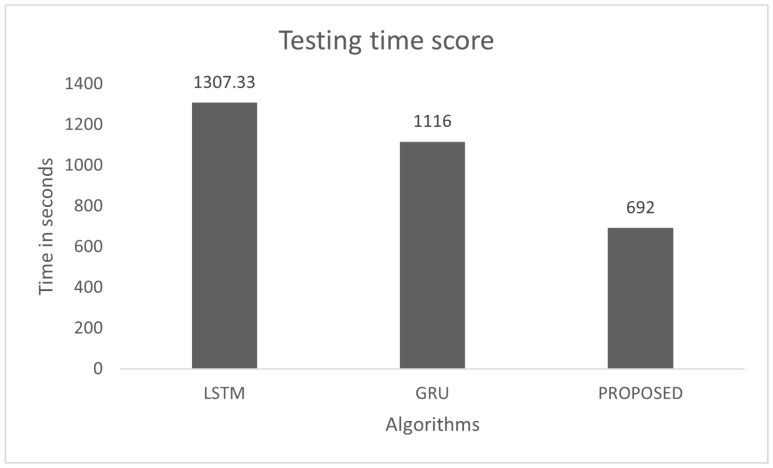
Testing Time on the combined DDoS dataset.

The model performance was tested for the response time on the combined DDoS dataset. Testing of the model was conducted with 20% of pre-processed data of the dataset. The time testing was is also conducted for three models—LSTM, GRU, and the proposed model. The proposed model was tested with 2,549,370 instances of the combined DDoS dataset, which were 79,668 batches of size 32. The total testing time was 692 s, as shown in Figure 13, and each batch took 8.7 ms. There were 32 instances in each batch and the response time of each instance was 0.27 ms. The total testing times of LSTM and GRU for the same instances were 1307.33 and 1116 s, respectively. The testing response time shows that the proposed model is faster than LSTM and GRU.

### 5.4. Intra-Vehicular Network

In the intra-vehicle network, the internal smart devices of a vehicle communicate with each other and control the communication of the vehicle in the inter-network. The hacker can target the internal network of the vehicle, called CAN bus. After gaining access to the intra-vehicular network, the assailant can manipulate and erase information, disrupt the vehicle functionalities, and can take control of the vehicle [39]. An internal attack on the car can cause accidents. In a fuzzing attack, the attacker shakes the steering wheel, changes gears, turns the signal lights on/off randomly, and uses the brakes in the mobility of the vehicle [33,48]. C. Miller and C. Valasek made two attacks on a CAN bus. The operation took place on an empty road in the country. In the first attack, they activated the auto parking property when the car jerked the steering wheel side to side of the road. In the second attack, they disabled the brakes while the vehicle was in motion. Both attacks did not cause any real damage, but if these stunts were performed in a crowded place it could lead to an accident [12].

**Figure 14:**
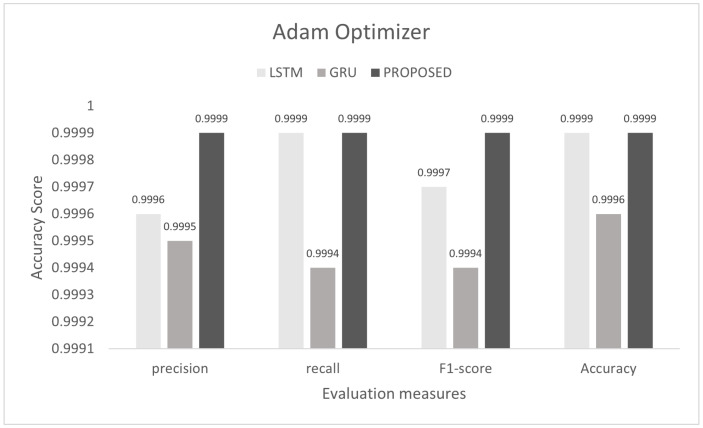
Evaluation on Adam with the car-hacking dataset.

**Figure 15:**
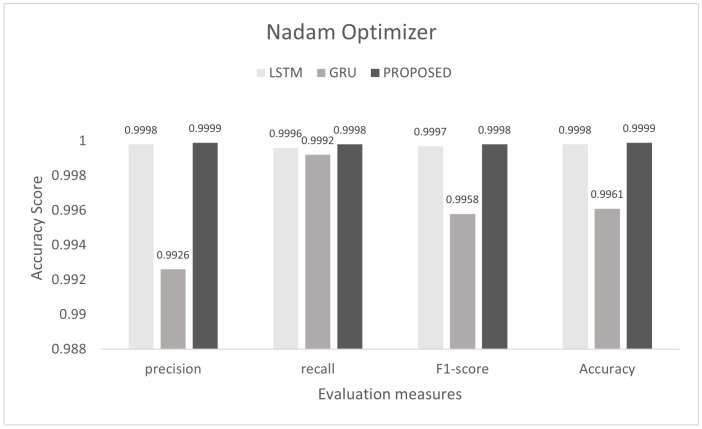
Evaluation on Nadam with the car-hacking dataset.

**Figure 16:**
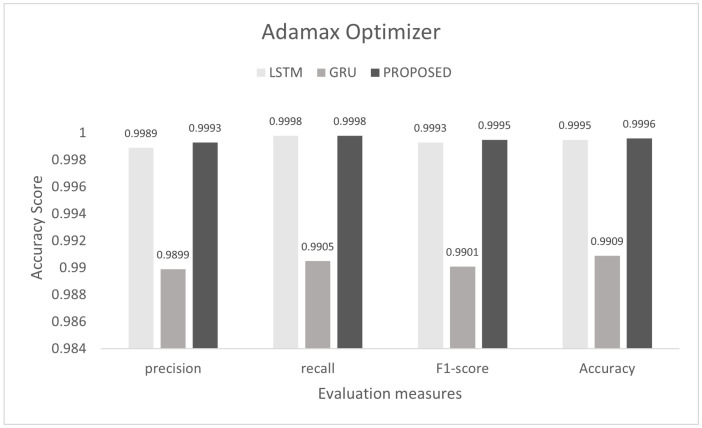
Evaluation on Adamax with the car-hacking dataset.

**Figure 17:**
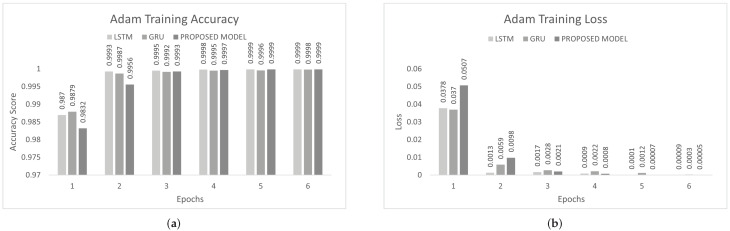
Adam training on the car-hacking dataset. (**a**) Adam training accuracy on car-hacking. Dataset; (**b**) Adam training loss on car-hacking. Dataset.

**Figure 18:**
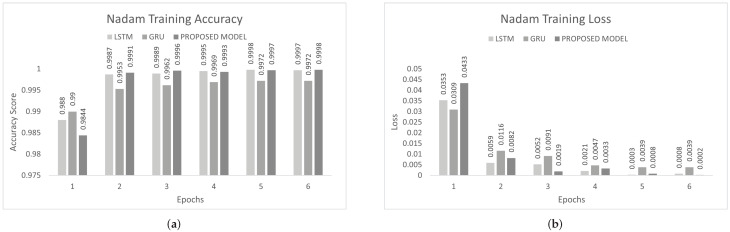
Nadam Training on the car-hacking dataset. (**a**) Nadam training accuracy on car-hacking. dataset; (**b**) Nadam training loss on car-hacking. dataset.

**Figure 19:**
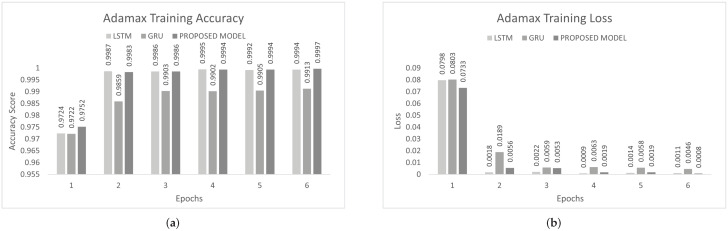
Adamax training on car-hacking dataset. (**a**) Adamax training accuracy on car-hacking. dataset; (**b**) Adamax training loss on car-hacking. dataset.

To control this type of accident, we conducted he experiment on the proposed model to detect multi-class malicious attacks in the internal network of the vehicle by using a car-hacking dataset. The proposed scheme was tested on Adam, Nadam, and Adamax optimizers. For multi-class, we used the softmax activation function in the last layer. The training process was conducted for six epochs. In this experiment, we used the sparse categorical crossentropy function for loss. The evaluation of the model for the intra-vehicle network was conducted with a car-hacking dataset. The precision, recall, F1-measure, and accuracy on Adam was 0.9999, 0.9999, 0.9999, and 0.9999, respectively. The precision, recall, F1-measure, and accuracy on the Nadam optimizer was 0.9999, 0.9998, 0.9998, and 0.9999, respectively. The precision, recall, F1-measure, and accuracy on the Adamax optimizer was 0.9993, 0.9998, 0.9995, and 0.9996, respectively. The comparisons with LSTM and GRU are shown in Figure 14, Figure 15 and Figure 16. The proposed model training accuracy and loss for Adam, Nadeem, and Admax optimizers are shown in Figure 17, Figure 18 and Figure 19, respectively. Training accuracy was over 99% and loss was below 0.02 for each optimizer.

**Figure 20:**
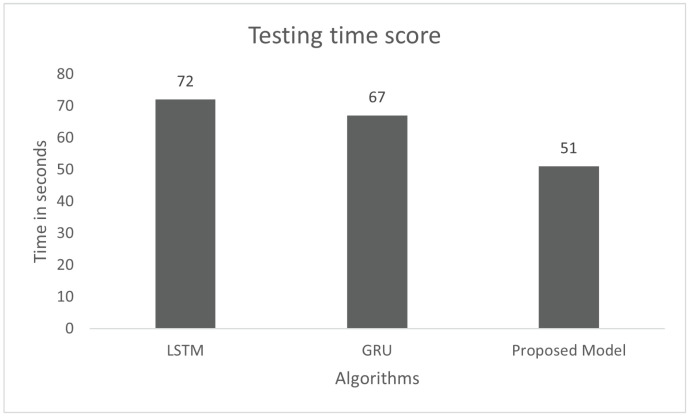
Testing time score on the car-hacking dataset.

The model performance was tested for the response time on the car-hacking dataset. Testing of the model was conducted with 20% of the pre-processed data of the dataset. The time testing process was also conducted for three models, such as LSTM GRU and the proposed model. The proposed model was tested with 1,001,720 instances of the dataset, which are 31,304 batches of size 32. The total testing time was 51 s, as shown in Figure 20, and each batch took 1.62 ms. There were 32 instances in each batch and the response time of each instance was 0.05 ms. The total testing time of LSTM and GRU for the same instances were 72 and 67 s, respectively. The testing response time shows that the proposed model is faster than LSTM and GRU.

### 5.5. Discussion

The experimental results show that the proposed model gives better performance in less response time as compared to other models. The proposed scheme can detect both binary and multi-class cyber attacks in less response time. We observed the proposed model with different optimizers for both datasets. The results show different values for each optimizer. The Adam and Nadam optimizers give better results than the Adamax optimizer. In this experiment, we obtained the highest results with Nadam on the proposed model and LSTM for both binary and multi-classification. In this work, we used k-fold cross-validations to test the performance of the proposed system. Moreover, 3-fold, 5-fold, and 7-fold for both DDoS and car-hacking datasets were used. The performance of the proposed system remained the same and the results are tabulated in Table 4 and Table 5. Comparison of the proposed study with other ML algorithms are highlighted in Table 4 and Table 5. The performance of LSTM is equivalent to the proposed model, but the response time is high, while the GRU model gives less performance than LSTM and the proposed model. The combination of LSTM and GRU with the ReLU DENSE layer gave a quick response. Deep layers of LSTM and GRU gave better results, but were time consuming. From the experimental results, the propose model gave above 99% results for binary and multi-class classification.

## 6. Conclusions

The extensive growth of smart vehicular networks has opened up several doors for cybercriminals. Attacks on intra-vehicle networks can cause deaths and severe accidents. This research proposes a hybrid DL-based model for intrusion detection in IoV. The proposed scheme contains a hybrid combination of LSTM and GRU that reduces the training and response time. The performance of the proposed approach was evaluated by conducting extensive experiments on a combined dataset of CIC DoS 2016, CICIDS 2017, and CSE-CIC-IDS 2018, and car-hacking datasets. The experimental results demonstrate that the proposed model achieves 99.5% accuracy for the combined DDoS dataset and 99.9% for the car-hacking dataset, respectively.

## Figures and Tables

**Table 1:** A comparison of existing studies related to intrusion detection in IoV.

Authors	Inter-Vehicle Detection	Intra-Vehicle Detection	Multiclass Detection	Features Filtering	Response Time
Ashraf et al. [34]	√	√	×	×	High
Injadat et al. [35]	√	×	×	√	Low
Zaidi et al. [36]	√	×	×	×	High
Anbalagan et al. [37]	√	×	×	×	Low
Nie et al. [38]	√	×	×	√	High
Olufowobi et al. [39]	×	√	×	×	Low
Zhang et al. [40]	×	√	√	×	Low
Kang et al. [41]	×	√	√	×	Low
Proposed Study	√	√	√	√	Low

**Table 2:** Comparison of the HDL-IDS with the state-of-the-art models.

Authors	Dataset	Attack Detection Mechanism
Ashraf et al. [34]	Car Hack and UNSWNB15	LSTM
Injadat et al. [35]	CICIDS 2017 and UNSW-NB 2015	NIDS
Anbalagan et al. [37]	Network traffic	ANN base SD-IoV
Nie et al. [38]	Network traffic	CNN
Olufowobi et al. [39]	CAN	SAIDuCANT
Zhang et al. [40]	NSL-KDD	Deep Belief Network (DBN)
Kang et al. [41]	CAN	DBN
Proposed Study	Combined DDoS and Car Hack2020	HDL-IDS

**Table 3:** The utilized datasets.

Dataset	Dataset-Files	Classes	Records
Combined DDoS	DDoS balanced	DDoS	6,472,647
		Benign	6,321,980
Car hack 2020	DDoS	Injected	587,521
		Normal	3,047,062
	Fuzzy	Injected	491,847
		Injected	3,259,177
	Spoof	Injected	1,252,149
		Injected	7,731,054
	Normal	Injected	0
		Injected	988,872

**Table 4:** Comparison of the HDL-IDS with other ML algorithms on the combined DDoS dataset.

Algorithms	Precision	Recall	F1-Score	Accuracy
Naive Bayes	0.8928	0.8924	0.8925	0.8925
Decision tree	0.9907	0.9750	0.9827	0.9814
SVM	0.9493	0.9125	0.9305	0.9302
LSTM	0.9925	0.9949	0.9937	0.9936
GRU	0.9819	0.9874	0.9846	0.9844
Proposed study	0.9951	0.9960	0.9952	0.9951

**Table 5:** Comparison of the HDL-IDS with other ML algorithms on the car-hacking 2020 dataset.

Algorithms	Precision	Recall	F1-Score	Accuracy
Naive Bayes	0.7903	0.7681	0.7790	0.7192
Decision tree	0.9503	0.8099	0.8745	0.9181
SVM	0.9379	0.8966	0.9167	0.9254
LSTM	0.9994	0.9997	0.9995	0.9997
GRU	0.9940	0.9963	0.9951	0.9952
Proposed Study	0.9997	0.9998	0.9997	0.9998

## Data Availability

The publicly available data set can be found at: https://ocslab.hksecurity.net/Datasets/CAN-intrusion-dataset and https://www.kaggle.com/devendra416/ddos-datasets.
